# Excretion Dynamics of Arboviruses in Mosquitoes and the Potential Use in Vector Competence Studies and Arbovirus Surveillance

**DOI:** 10.3390/tropicalmed8080410

**Published:** 2023-08-11

**Authors:** Christin Körsten, Ana Vasić, Amira A. AL-Hosary, Birke A. Tews, Cristian Răileanu, Cornelia Silaghi, Mandy Schäfer

**Affiliations:** 1 Institute of Infectology, Friedrich-Loeffler-Institut, Federal Research Institute for Animal Health, Greifswald-Insel Riems, 17493 Greifswald, Germany; christin.koersten@fli.de (C.K.);; 2 Scientific Institute of Veterinary Medicine of Serbia, 11000 Belgrade, Serbia; 3 Department of Animal Medicine (Infectious Diseases), Faculty of Veterinary Medicine, Assiut University, Assiut 71526, Egypt

**Keywords:** mosquito excreta, arbovirus, surveillance, vector competence, West Nile virus, Usutu virus, tick-borne encephalitis virus

## Abstract

The increasing threat of arboviruses such as West Nile virus (WNV) and Usutu virus (USUV) requires the fast and efficient surveillance of these viruses. The examination of mosquitoes takes up an important part; however, these investigations are usually very time-consuming. An alternative sample type for arbovirus surveillance might be mosquito excreta. In order to determine the excretion dynamics under laboratory conditions, laboratory colonies of *Aedes vexans* and *Culex pipiens* biotype *molestus* were infected with WNV, USUV or tick-borne encephalitis virus (TBEV). After infection, the excreta were sampled and investigated for viral RNA. Excretion of viral RNA together with infectious blood meal could be detected up to five days after infection. Further excretion seemed to correlate with a disseminated infection in mosquitoes, at least after USUV infection. In addition, it could be determined that the amount of viral RNA in the excretions correlated positively with the viral load in the mosquito bodies. Overall, this study shows that the usage of mosquito excreta as a sample type for surveillance enables the detection of endemic viruses (WNV, USUV) as well as non-mosquito-borne viruses (TBEV). In addition, examination of viral shedding during vector competence studies can provide insights into the course of infection without sacrificing animals.

## 1. Introduction

In past decades, European countries have repeatedly experienced the (re-)emergence of arthropod-borne viruses (arboviruses) [1,2,3]. In Germany, the flaviviruses (genus *Flavivirus*, family *Flaviviridae*) West Nile virus (WNV) and Usutu virus (USUV) have become endemic in recent years. USUV is distributed throughout the country, while the distribution of WNV is currently limited to the eastern part of the country [4]. Infections of these two viruses in humans, birds and horses are reported annually [4,5,6]. Both viruses pose a threat to human and animal health [7,8], and they might also affect the safety of blood donations [9]. Thus, it is urgently necessary to control and combat these viruses.

In order to control circulating arboviruses like WNV and USUV, effective and fast monitoring is essential [3,10]. There are various methods that can be used for arbovirus surveillance. For the purpose of sensitive and fast monitoring, the One Health approach, in which all the potential hosts and vectors are monitored, has proven itself [11,12,13]. Mosquitoes, as vectors, play a particularly important role in an early warning system. During an outbreak of WNV in Spain, for example, the first infected mosquitoes were detected one month before the first human cases occurred [14]. Furthermore, in Germany, the emergence of USUV was first noticed when an infected mosquito was found [15].

For the surveillance of WNV and USUV in mosquito populations in Germany, adult mosquitoes are usually caught with traps and taken to the laboratory, where the species is determined based on the morphological features or molecular biological markers. Then, the specimens are pooled and examined for viral genomes using molecular methods [16,17]. However, there are some limitations in mosquito surveillance. Infection rates in field-collected mosquitoes are usually very low, as most traps are targeted at trapping host-seeking females [11,18]. Both aforementioned German surveillance studies [16,17] investigated a large number of mosquito pools (445 and 4144, respectively), although the viral genomes of WNV and USUV were detected in only a few. Therefore, a large number of individuals have to be examined for successful surveillance. However, these investigations are very time-consuming and require a high level of lab staff and material. Constant improvement in mosquito surveillance is necessary to guarantee effective and fast surveillance.

In recent years, an alternative surveillance method has been developed, which uses sugar-baited Flinders Technology Associates (FTA)^TM^ cards to collect mosquito saliva. This method has already proven to be effective for the detection of various arboviruses [19,20,21]. In Germany, WNV and USUV have already been detected using this method [22,23]. Thus, collecting mosquitoes for surveillance is no longer absolutely necessary. Another sample type that can be collected from mosquitoes without trapping them is their excretions. It was already shown that it is possible to detect the viral genomes of WNV, DENV and Ross River virus (genus *Alphavirus,* family *Togaviridae*) in the excreta of infected mosquitoes [24,25]. Moreover, the application of mosquito excreta in field surveillance has been tested and proved to be effective [26,27,28]. In addition to arboviruses, it is also possible to detect other blood-borne pathogens in mosquito excreta [29,30]. Last but not least, there is also the opportunity of detecting non-mosquito-borne pathogens in mosquito excreta in cases where the mosquitoes fed on an infected host before [29,31]. Compared to saliva samples, mosquito excreta have a higher positivity rate, also in non-vector-competent mosquito species, making the use of this sample type in mosquito surveillance even more sensitive [24].

In addition to the application in arbovirus surveillance, mosquito excreta can also be used in future vector competence studies. So far, examination of mosquito excreta in vector competence studies has not been used, as only recently the potential of this sample type has been explored in studies. These studies found that the excretion of viral RNA seems to correlate with the viral dissemination in the mosquito vector [24,25]. Examination of the excreta after an experimental infection can therefore provide information about the infection status of the mosquito without the need to sacrifice it. Furthermore, this non-destructive method also provides the opportunity to study the course of infection in a mosquito [25].

Against the background of WNV and USUV circulation in Germany, and given the potential emergence of further arboviruses [32], examination of mosquito excreta represents an attractive alternative for arbovirus surveillance as well as vector competence research. However, until now, most of the studies that investigated the arboviral excretion of mosquitoes have been conducted outside of Europe. In this study, we investigated the excretion dynamics of USUV, WNV and the non-mosquito-borne tick-borne encephalitis virus (TBEV; genus *Flavivirus*, family *Flaviviridae*) in common German mosquito species. The results of these investigations enable the use of this sample type for arbovirus surveillance and research in Central Europe.

## 2. Materials and Methods

### 2.1. Cells and Viruses

All the cell lines that were used were provided by the biobank of the Friedrich-Loeffler-Institut. Vero-B4 cells were used for the propagation of WNV and for the vector competence studies with WNV and USUV. Vero-76 cells were used for the propagation of USUV. For TBEV, A549 cells were used for the virus propagation, and HEK-293 cells were used for the vector competence studies. A list of the viruses used in this study can be found in Table 1.

### 2.2. Mosquito Infection and Examination of Vector Competence

Laboratory colonies of *Culex pipiens* biotype *molestus* (Hesse, Germany, 2002) and *Aedes vexans* “Green River” (UT, USA, 2000) were used for all the infection experiments. Rearing of these mosquitoes as well as the implementation of the vector competence studies were performed as previously described [33]. In summary, the mosquitoes were offered an infectious blood meal via cotton stick feeding, and the engorged females were sorted into incubation chambers either individually or in groups of five. The females were then incubated for 14 or 20 days under controlled conditions (26 °C, 85% humidity, 16:8 light–dark photocycle). After incubation, all the surviving mosquitoes were dissected and forced to salivate [34].

### 2.3. Collection of Mosquito Excreta

**Figure 1:**
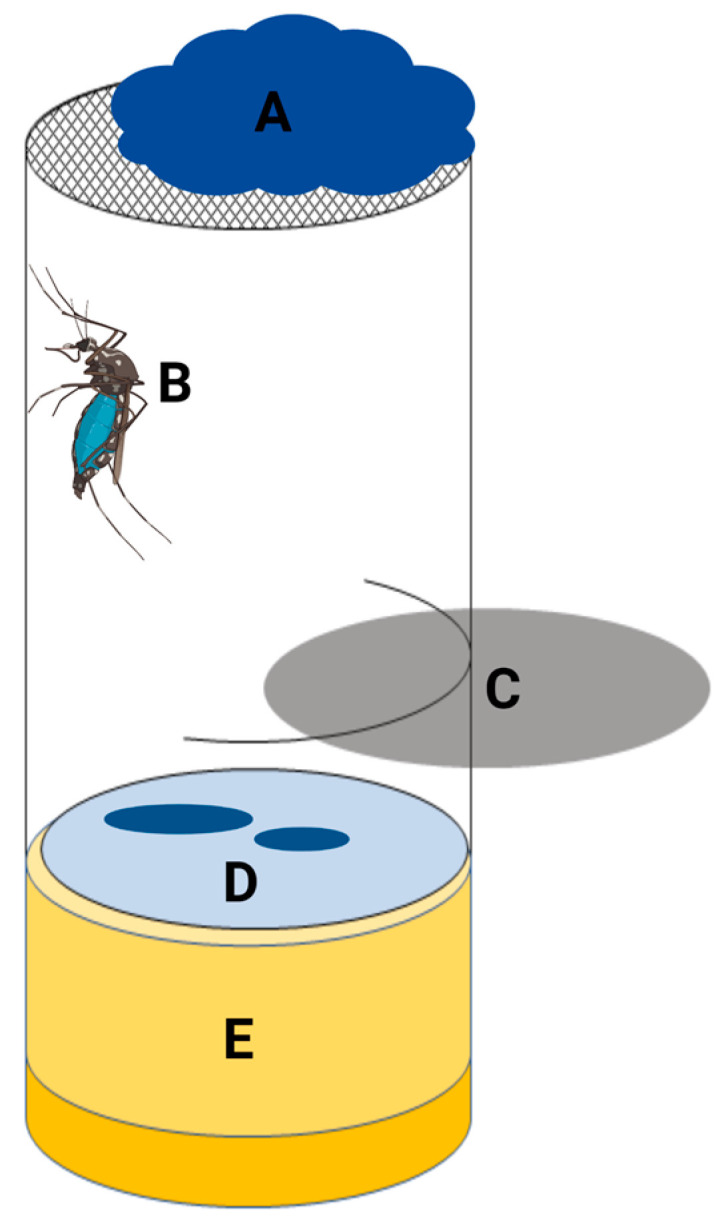
Incubation chamber for the collection of mosquito excreta. (A) Cotton pad soaked with blue-colored sugar placed on mosquito net; (B) mosquito fed on blue-colored sugar; (C) insert to separate the mosquitoes during the changing of the FTA cards or Parafilm; (D) FTA cards or Parafilm with mosquito excreta; and (E) plug. Created with BioRender.com.

For the collection of the mosquito excreta, either Whatman^TM^ FTA^TM^ classic cards (Cytiva, Marlborough, MA, USA) or pieces of Parafilm M (Bemis, Neenah, WI, USA) were used. The FTA cards or pieces of Parafilm were placed on the bottom of the incubation chambers. In order to prevent the mosquitoes from escaping while changing the FTA cards or Parafilm, the chambers were prepared in such a way that a disk could be inserted to separate the mosquitoes in the upper part of the chamber. To ensure the visibility of the mosquito excreta, the mosquitoes were offered blue-colored (indigo carmine; Carl Roth, Karlsruhe, Germany) sugar solution (Figure 1).

The FTA cards and Parafilm were sampled regularly during the incubation period. Whenever possible, samples were taken daily, excluding weekends and public holidays. During the first two experiments, both FTA cards and Parafilm were used. In the case of the FTA cards, all the colored spots were cut out and placed into 2 mL tubes (Eppendorf, Hamburg, Germany) filled with 560 µL AVL buffer with carrier RNA (Qiagen, Hilden, Germany). The Parafilm was wiped with a cotton stick soaked with 1 × phosphate buffer saline (PBS), and then the cotton stick was placed into a 2 mL tube with 500 µL PBS. It was possible to detect viral RNA in the excreta collected from the FTA cards and Parafilm (Figure S1). Overall, taking samples using Parafilm appeared to be handier and easier. Therefore, for all further experiments, only Parafilm was used. In order to achieve a greater stability of viral RNA, AVL buffer was used instead of 1 × PBS, and the cotton sticks were placed into 2 mL tubes filled with 560 µL AVL buffer.

**Figure 2:**
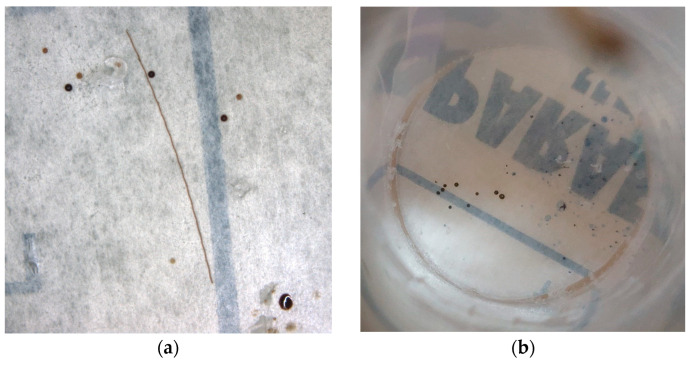
Macroscopic properties of mosquito excreta on Parafilm. (**a**) Dark red shedding during the first four days; and (**b**) dark blue shedding due to the coloring of the sugar solution.

After each sampling, new FTA cards or pieces of Parafilm were placed into the incubation chambers. Based on the results of the first experiments, samples were only taken in further trials when the mosquito excreta were visible, as shown in Figure 2. If no excretion was visible, the Parafilm was not changed.

A summary of all the infection experiments can be found in Table 2. During all the experiments, except for experiments #1 and #2, engorged females that died during the incubation period were sampled in 560 µL AVL buffer.

### 2.4. Nucleic Acid Extractions and Analysis

Processing of the samples in preparation for the extractions was performed as already described [33]. Nucleic acids were extracted with the NucleoMag VET Kit (Macherey-Nagel, Düren, Germany) according to the manufacturer’s instructions in a KingFisher Flex (ThermoFisher Scientific, Darmstadt, Germany) or a BioSprint 96 (Qiagen). Then, 1 µL of an internal control [35] was added to each sample in order to ensure the success of the extraction.

Molecular investigation was performed using a multiplex RT-qPCR in a CFX96 Real-Time PCR detection system (Bio-Rad Laboratories, Feldkirchen, Germany). The detection of viral RNA was performed with specific primers and probes that detected the WNV 5′ nontranslated region [36], the USUV nonstructural protein 1 region [15] and the TBEV 3′ nontranslated region [37]. Detection of the internal control was performed with specific primers and probes [35] during each RT-qPCR. The composition of the master mix and the conditions of the RT-qPCR for the detection of WNV and USUV RNA have been described previously [33,38]. For the detection of TBEV RNA, the iTaq™ Universal Probes One-Step Kit (Bio-Rad Laboratories) was used, together with 1.0 µL of each primer (10 µM), a 0.5 µL probe (10 µM) and a 5 µL sample in a total volume of 20.0 µL.

Analysis of the results was performed with the Bio-Rad CFX Maestro Software, Version 1.1 (Bio-Rad Laboratory). Relative quantification of the WNV and USUV RNA was performed with a standard curve based on a 10-fold dilution series of the used virus stocks. A cut-off quantification cycle (Ct) value of 36.00 was determined for the WNV and USUV PCR assays based on previous results [33]. The same cut-off was also used for the TBEV PCR assay based on the results of a 10-fold dilution series.

### 2.5. Vector Competence Indices and Infection Status

The vector competence indices were defined as in [33,38]. The infection rate (IR) is the proportion of mosquitoes with an infection in their bodies out of all the mosquitoes that were investigated. The dissemination rate (DR) is the proportion of mosquitoes with viral RNA in their legs and wings out of all the mosquitoes with an infected body. The transmission rate (TR) is the proportion of mosquitoes with potential transmission (i.e., viral RNA or infectious virus in their saliva) out of all the mosquitoes with a disseminated infection. The infection status was defined on the basis of these indices, as designated by infection (corresponds to the IR), disseminated infection (corresponds to the DR) and potential transmission (corresponds to the TR).

### 2.6. Statistical Analysis

Statistical analysis was performed with SigmaPlot11 (Systat Software, Version 11, Düsseldorf, Germany). Evaluations were performed for experiments #6 and #7, since all the other experiments had a low infection rate.

Student’s *t*-test or the Mann–Whitney rank sum test was used to compare the amount of viral RNA in the mosquito excreta depending on the infection status. The mean values of the amount of viral RNA in the mosquito excreta collected over the entire incubation period, from the 5th day post infection (dpi) and from the 7th dpi, were used for these evaluations. Mosquitoes that died after the 12th dpi were also included in these calculations.

The Pearson product–moment correlation test was used to examine the correlation between the amount of viral RNA in the excreta and the viral load in the mosquito bodies or legs and wings. To test the correlation between the viral shedding and viral load in the mosquito bodies or legs and wings, respectively, only data from mosquitoes that survived the incubation period and were dissected were used.

A statistical difference was assumed at a *p* value of ≤0.05. A strong correlation was assumed at an *r* value of >0.5; a medium correlation was assumed at an *r* value from 0.1–0.5; and a weak correlation was assumed at an *r* value of <0.1.

## 3. Results

### 3.1. Vector Competence Trials

During all the infection experiments, the feeding and survival rates of the mosquitoes were determined (Table S1). In addition, for each infection experiment, the blood meal titer was determined to demonstrate a successful infection. Moreover, the IR, DR and TR were determined to assess the vector competence. The results of the blood meal titrations and the vector competence indices are shown in Table 3.

There was no large drop in the blood meal titers during the blood feedings. However, for unknown reasons, in experiment #5, the blood meal titer was one log TCID_50_/mL lower than calculated. In order to avoid further problems, a different USUV strain was used for the other USUV infection.

Overall, the *Ae. vexans* laboratory colony “Green River” appeared to be not susceptible to both tested WNV lineages. In contrast, the *Cx. pipiens* biotype *molestus* laboratory colony was vector-competent for WNV lineage 2 as well as for USUV lineage Europe 3 after infection with a higher blood meal titer (Tables S2–S4). The vector competence of this mosquito species for WNV lineage 1 could not be determined as only two mosquitoes were investigated. In addition, none of the tested *Cx. pipiens* biotype *molestus* mosquitoes were found to be infected after oral infection with TBEV.

### 3.2. Excretion of Viral RNA via the Infectious Blood Meal

In order to determine how long viral RNA is excreted together with the infectious blood meal, two experiments were carried out with a mosquito-borne flavivirus in a non-vector competent species (WNV in *Ae. vexans*; experiment #3) and with a non-mosquito-borne flavivirus (TBEV in *Cx. pipiens* biotype *molestus*; experiment #4).

**Figure 3:**
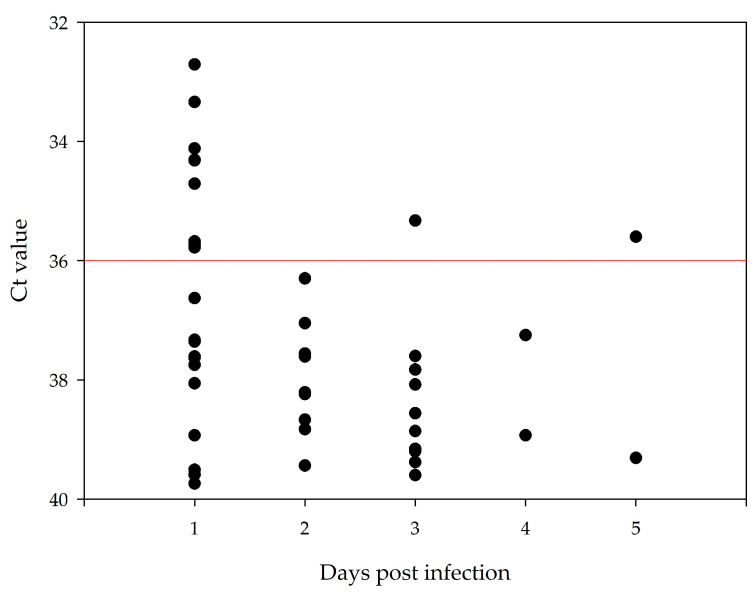
Ct values measured from mosquito excreta after infection with TBEV in *Cx. pipiens* biotype *molestus* (experiment #4). The red line represents the selected cut-off at a Ct value of 36.00. After the 5th day, no positive signal was detected.

The Ct values measured in the excreta of *Ae. vexans* were always above 36.00 and thus above the selected cut-off. In addition, these high Ct values were only detected up to 4 days after infection. In the *Cx. pipiens* biotype *molestus* infected with TBEV, excretion of viral RNA below the selected cut-off could be detected up to 5 days after infection. Figure 3 displays the measured Ct values in the excreta from TBEV infected mosquitoes from the 1st to 5th dpi.

Taken together with the macroscopic observations that dark red spots were observed during the first four days (Figure 2a), it can be assumed that the excretion of viral RNA together with the infectious blood meal can be detected up to five days after infection.

### 3.3. Dependence of Viral Shedding on Mosquito Infection Status

**Figure 4:**
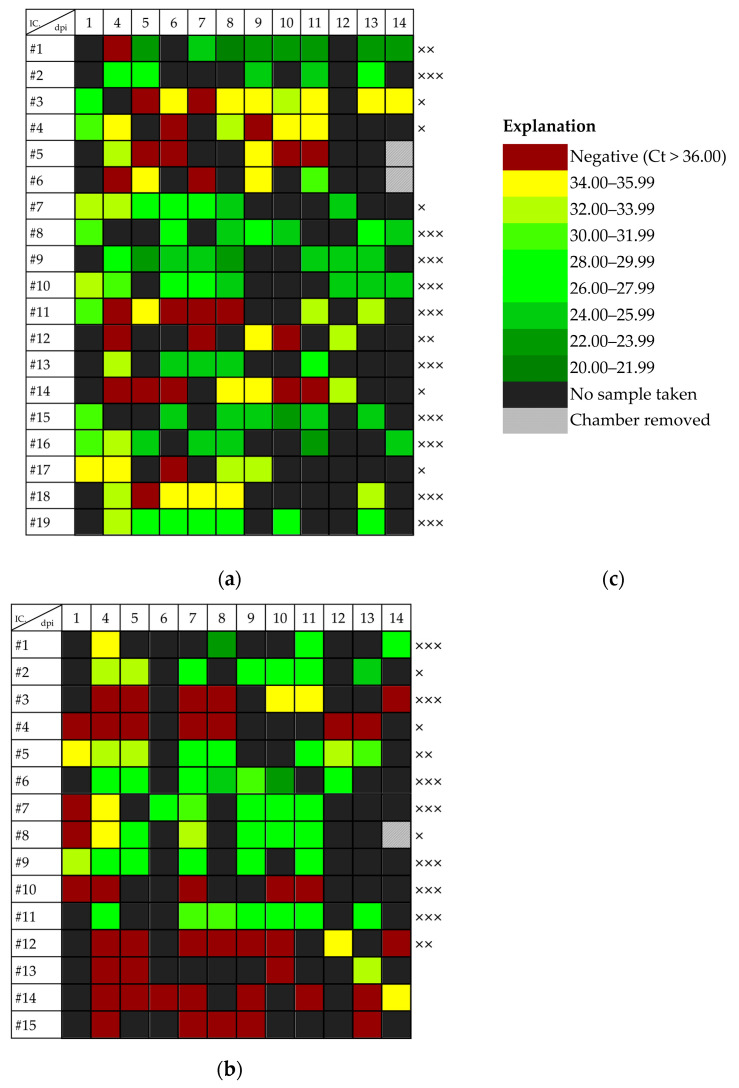
Excretion of viral RNA after infection with (**a**) USUV lineage Europe 3 (Germany, 2011), experiment #6 and (**b**) WNV lineage 2 (Germany, 2018), experiment #7. Only data from mosquitoes that survived the 12th day post infection (dpi) are shown. The infection status of the individual mosquitoes is indicated as infection (×), disseminated infection (××) and potential transmission (×××). (**c**) Colors indicate the measured Ct values in the mosquito excreta collected from each incubation chamber (IC).

Since the excretion of viral RNA together with the infectious blood meal was demonstrated up to the 5th dpi, a question remained regarding to what extent the viral shedding after 5 dpi is associated with an infection in the mosquitoes. Excretion of viral RNA after the 5th dpi could be detected in almost all the infected mosquitoes (Figure 4 and Figure S2, Tables S2–S4). Figure 4 displays the Ct values measured in the excretions and the infection status of individual mosquitoes after infection with USUV or WNV. The greatest amount of viral RNA was excreted by a mosquito with a disseminated USUV infection on the 8th dpi (Ct value of 20.76; corresponds to a titer equivalent of 6.58 × 10^5^ TCID_50_/mL). However, in two mosquitoes with WNV infection (one infection, one potential transmission), there was no detectable excretion of viral RNA during the entire incubation period (Figure 4b, Table S4). On the other hand, viral excretion after the 5th dpi was detected in a total of 11 non-infected animals in experiments #5, #6 and #7 (2 × WNV, 9 × USUV; Figure 4 and Figure S2, Tables S2–S4).

In order to determine whether and to what extent viral shedding depends on the infection status of mosquitoes, the amount of viral RNA in the excretion was examined in relation to the infection status of individual mosquitoes. For the WNV infection, no statistical differences in the amount of viral excretion depending on the infection status could be found. For the USUV infection, no statistical differences were found between infected and non-infected mosquitoes. However, the amount of viral RNA in the excretion during all the tested periods was significantly higher in the mosquitoes with a disseminated USUV infection compared to the mosquitoes with a non-disseminated infection (*p* = 0.006, 0.034, and 0.013 from the 1st, 5th and 7th dpi, respectively). Similarly, there was also a significant difference depending on potential transmission (*p* = 0.049, 0.034, and 0.027 from the 1st, 5th and 7th dpi, respectively) (Table S5).

### 3.4. Correlation between Amount of Viral RNA in Excreta and Viral Load in Mosquitoes

**Figure 5:**
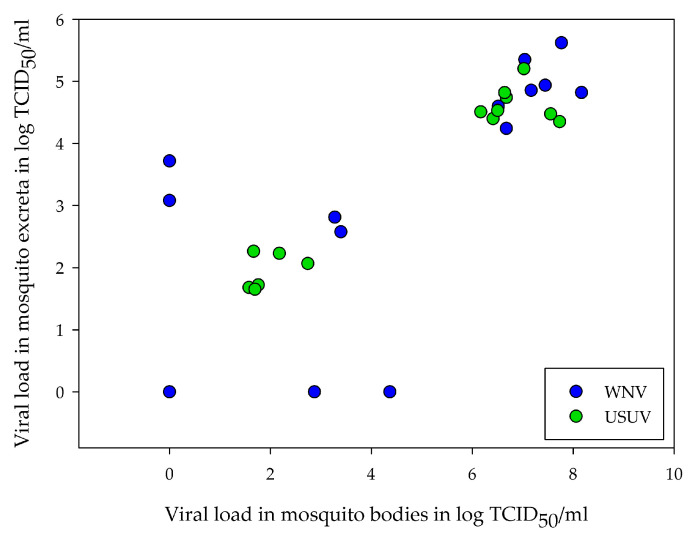
Correlation between the amount of viral RNA in the mosquito excreta collected from the 7th dpi and the viral load in the mosquito bodies after infection of *Cx. pipiens* biotype *molestus* with USUV (experiment #6) and WNV (experiment #7).

In addition to the dependency on the infection status, a possible correlation between the viral loads in the excreta and the mosquito bodies or legs and wings was also investigated. Overall, there was a positive correlation between the amount of viral RNA detected in the excreta and the amount of viral RNA detected in the mosquito bodies. This correlation was found during all the tested time periods for the mosquitoes infected with WNV (*p* = 0.0166, 0.0110, and 0.0097 from the 1st, 5th and 7th dpi, respectively) and USUV (*p* ≤ 0.001 for all the tested time periods). In all cases, a strong correlation based on the *r* value was found (*r* > 0.5; Table S6). Figure 5 shows an example of the correlation between the body loads and the viral loads in the excreta collected from the 7th dpi for the WNV and USUV infected mosquitoes. In contrast, the amount of viral RNA in the excreta did not correlate with the viral load in the legs and wings (Table S7).

### 3.5. Duration of Viral Shedding after Infection

The duration of viral shedding from infected mosquitoes is decisive for the use of mosquito excreta in experiments or surveillance. Infected mosquitoes usually excreted high amounts of viral RNA up to the last day of the incubation period (14th dpi). In experiment #2, it was also possible to detect viral RNA in the excreta up to the last day of the incubation period (20th dpi; Figure S1); however, the infection status of these mosquitoes was not determined since these animals died before dissection and forced salivation on the 20th dpi.

## 4. Discussion

Examination of mosquito excreta for viral RNA could be a valuable tool for future arbovirus surveillance and vector competence studies. The aim of this study was to investigate the excretion dynamics of German mosquito species for arboviruses circulating in Germany (WNV, USUV, TBEV) in order to determine a possible use of this sample type in arbovirus research and monitoring.

First, two different materials for collection (FTA cards and Parafilm) were tested and compared in terms of their efficiency and applicability under laboratory conditions. With both materials, it was possible to detect viral RNA in mosquito excreta without any obvious differences in quality and quantity. Another study comparing FTA cards and polycarbonate for the sampling of mosquito excreta also found a comparable quantity of viral RNA after 24 h [39]. As Parafilm appeared to be a cost-effective alternative and more convenient to use than FTA cards, this material is suitable for the frequent sampling required during vector competence studies. In fact, Parafilm or other plastic material has already been used in other studies examining viral shedding in mosquitoes [24,28]. However, compared to plastic material, FTA cards have the advantage that the collected viral RNA remains stable for a longer period of time [39]. Therefore, the use of Parafilm for arbovirus surveillance is still questionable and depends on the envisaged sampling frequency.

During the first four days, the collected excreta were mostly dark red in color, probably indicating the excretion of the blood meal. This observation is in accordance with other studies reporting dark red spots during the first three days after infection, which correlates with the blood digestion time in mosquitoes [24,25,40]. In the current study, the potential excretion of viral RNA together with the blood meal was observed even after the third day. The latest detection of viral RNA probably excreted together with the blood meal was five days after infection, after an oral infection with the non-mosquito-borne TBEV in *Cx. pipiens* biotype *molestus.* The duration of the excretion of the blood meal might also depend on the amount of ingested blood and the ingested viral dose. In almost all the experiments, the blood meal titer was between 1 × 10^6^ and 1 × 10^7^ TCID_50_/mL, which corresponds to a natural infection dose in viremic WNV-infected birds [41,42]. It can therefore be stated that viral RNA can be excreted up to five days after infection together with the infectious blood meal, even by non-infected mosquitoes.

Almost all the infected mosquitoes excreted a large quantity of viral RNA over the entire incubation period. After an infection with USUV, the excretion of viral RNA seemed to depend on whether the mosquitoes had a disseminated infection. Similar results were also observed by Fontaine et al. [25] after infection with DENV and by Ramirez et al. [24] after infection with WNV and Ross River virus. In addition, there was a correlation between the viral load in the mosquito body and the amount of viral RNA in the excreta. A higher virus dose in the mosquito midgut can facilitate overcoming the midgut escape barrier and thus promote a disseminated infection [43]. Therefore, a higher viral load in the mosquito midgut could lead to a higher viral load in the excreta and, at the same time, to a higher dissemination rate. This could explain why the quantity of viral RNA in excreta depends on the dissemination but not the infection rate.

In the case of the WNV infection, a correlation between the viral load in the mosquito body and in the mosquito excreta could be found as well. However, the dependency on the dissemination rate could not be determined, although another study was able to prove this correlation after WNV infection [24]. One explanation for this result could be the low proportion of non-infected animals. In addition, it appears that a (disseminated) infection in mosquitoes does not always lead to the excretion of viral RNA. Two mosquitoes with WNV infection or potential transmission did not shed viral RNA over the entire incubation period. However, both mosquitoes had a relatively low viral load in their bodies. Against the background of the correlation between the viral load in bodies and excreta, it is conceivable that the excretion of these mosquitoes may have been below the detection limit. In contrast, some animals that were not infected also excreted viral RNA after the fifth day after infection. A possible explanation could be an early viral replication in the midgut epithelium, which, however, is suppressed by the mosquito immune system at a later time point. In addition, some of these mosquitoes were collected dead from the chambers, so a degradation of viral RNA before sampling cannot be ruled out. However, in six non-infected mosquitoes that survived to the last day and were examined accordingly, viral excretion was detected even after 10 days after infection. In these cases, too, an early viral replication that is suppressed later is conceivable. Nevertheless, the possibility of contamination during the sampling cannot be ruled out. Samples were taken with reasonable care, although disinfectants were not used during sampling as this could have irritated the mosquitoes. A further improvement in the methodology to avoid possible contamination should therefore be considered.

In the current study, the excretion of viral RNA could be detected up to 20 days after infection. The mosquitoes from this experiment were not examined for their infection status, but it can be assumed that the excretion was caused by a viral replication in at least one mosquito since the Ct values decreased over time. Fontaine et al. [25] were able to detect the excretion of DENV RNA even up to 26 days after infection, before the experiment was terminated. Thus, viral excretion by infected mosquitoes can be detected at least for three weeks after the intake of an infectious blood meal, possibly even longer.

Overall, this study proves that the examination of mosquito excreta can be a valuable method for future arbovirus surveillance in Germany. Other studies have already demonstrated that the detection of mosquito-borne viruses in the excreta from field mosquitoes during surveillance is possible. L’Ambert et al. [26] detected WNV in France in excreta collected on filter paper, and Meyer et al. [27] found WNV, Ross River virus and Murray Valley encephalitis virus (genus *Flavivirus*, family *Flaviviridae*) in Australia in excreta collected on FTA cards. In Germany, the collection of mosquito excreta might be used for the detection of WNV and USUV, as shown in this study.

Another advantage of the usage of mosquito excreta in surveillance is the possibility to detect other pathogens that are not transmitted by mosquitoes. During surveillance in Ghana, a non-mosquito-borne pathogen was found in the excreta of field mosquitoes [29]. In the current study, too, it was possible to demonstrate the detection of TBEV RNA in mosquito excreta after the oral intake of this virus. TBEV is widespread in Germany [44,45] and may also be detected in the excreta from German mosquitoes, since mosquitoes may also act as free blood collectors in humans and animals [46].

However, there are some limitations when using this sample type in surveillance. Viral RNA is usually only stable to a limited extent when collected on Parafilm [39]. In addition, it is still necessary to carry out several PCR assays and use other molecular methods to detect other pathogens than WNV and USUV. A combination of other methods, for example, the use of monoclonal antibodies to detect viral proteins [47], is needed in order to ultimately conduct fast and efficient surveillance.

Moreover, this sample type might also be suitable for vector competence research. Since the amount of viral RNA in excreta provides an indication of the infection status and the viral load in the mosquito body, examining the excretion enables the investigation of the course of the infection without sacrificing animals. This could play a role, for example, when performing sequential co-infections. Investigation of excreta could be used to confirm a successful infection with the first pathogen before a second infection is carried out.

## 5. Conclusions

After oral infection with WNV, USUV or TBEV in mosquitoes, it was possible to detect the RNA of these viruses in the mosquito excreta. The excretion of viral RNA correlated with the viral load in the mosquito body. In addition, the amount of USUV RNA in the excreta depended on whether the mosquitoes had a disseminated infection. Therefore, mosquito excreta represent a useful sample type for future vector competence studies and arbovirus surveillance.

## Figures and Tables

**Table 1:** Virus strains used in the vector competence studies.

Virus Strain	GenBank Accession No.	Titer ^1^
WNV lineage 1 (Italy, 2008)	JF719066	5.62 × 10^7^
WNV lineage 2 (Germany, 2018)	MH924836	1.33 × 10^9^
USUV lineage Europe 3 (Germany, 2011)	HE599647	5.01 × 10^7^
USUV lineage Europe 3 (Germany, 2016)	KY084498	5.62 × 10^7^
TBEV Western Neudoerfl	U27495	1.00 × 10^8^

^1^ in 50% tissue culture infectious dose per mL (TCID_50_/mL).

**Table 2:** Overview of all the infection experiments in *Culex pipiens* biotype *molestus* (CxM) and *Aedes vexans* (AeV), and the method used for excreta collection.

Experiment No.	Virus	Mosquito Species	Incubation Period (Days)	Number of Mosquitoes per Incubation Chamber	Material Used for Excreta Collection
#1	WNV lineage 1 (Italy, 2008)	AeV	14	1 or 5	FTA cards; Parafilm and 1 × PBS
#2	WNV lineage 1 (Italy, 2008)	CxM	20	5	FTA cards; Parafilm and 1 × PBS
#3	WNV lineage 2 (Germany, 2018)	AeV	14	1	Parafilm and AVL buffer
#4	TBEV Western Neudoerfl	CxM	14	1	Parafilm and AVL buffer
#5	USUV lineage Europe 3 (Germany, 2016)	CxM	14	1	Parafilm and AVL buffer; only if excreta were visible
#6	USUV lineage Europe 3 (Germany, 2011)	CxM	14	1	Parafilm and AVL buffer; only if excreta were visible
#7	WNV lineage 2 (Germany, 2018)	CxM	14	1	Parafilm and AVL buffer; only if excreta were visible

**Table 3:** Blood meal titers and infection (IR), dissemination (DR) and transmission rates (TR) of *Culex pipiens* biotype *molestus* (CxM) and *Aedes vexans* (AeV) in the infection trials.

Experiment No.	Virus	Mosquito Species	Blood Meal Titer ^1^	Mosquitoes Examined ^2^	IR % (n/n)	DR % (n/n)	TR % (n/n)
#1	WNV lineage 1 (Italy, 2008)	AeV	6.125	10	0 (0/10)	N/A	N/A
#2	WNV lineage 1 (Italy, 2008)	CxM	5.500	2	0 (0/2)	N/A	N/A
#3	WNV lineage 2 (Germany, 2018)	AeV	7.438	8	0 (0/8)	N/A	N/A
#4	TBEV Western Neudoerfl	CxM	7.063	24	0 (0/24)	N/A	N/A
#5	USUV lineage Europe 3 (Germany, 2016)	CxM	6.063	18	5.56 (1/18)	0 (0/1)	N/A
#6	USUV lineage Europe 3 (Germany, 2011)	CxM	7.125	16	100.00 (16/16)	75.00 (12/16)	83.33 (10/12)
#7	WNV lineage 2 (Germany, 2018)	CxM	6.844	14	78.54 (11/14)	81.82 (9/11)	77.78 (7/9)

^1^ Mean value of titrations before and after feeding in log TCID_50_/mL. ^2^ Total number of mosquitoes that survived until the last day of the experiment and were dissected and forced to salivate accordingly.

## Data Availability

The data that support the findings of this study are available in the main manuscript and in the supplementary materials accompanying this article.

## Supplementary Materials

The following supporting information can be downloaded at https://www.mdpi.com/article/10.3390/tropicalmed8080410/s1, Figure S1: Comparison between the methods for collecting mosquito excreta. Mosquitoes from the same population were infected and excreta were collected on either Parafilm or FTA cards during the entire incubation period; Figure S2: Excretion of viral RNA after infection with USUV lineage Europe 3 (Germany, 2016) at a low titer; Table S1: Feeding and survival rates of *Culex pipiens* biotype *molestus* (CxM) and *Aedes vexans* (AeV) in the infection experiments; Table S2: Viral loads in the mosquito bodies, legs and wings and saliva, and the corresponding amount of viral RNA in the mosquito excreta collected from the 1st or 7th day post infection (dpi) with USUV lineage Europe 3 (Germany, 2016) in *Culex pipiens* biotype *molestus* (experiment #5); Table S3: Viral loads in the mosquito bodies, legs and wings and saliva, and the corresponding amount of viral RNA in the mosquito excreta collected from the 1st, 5th or 7th day post infection (dpi) with USUV lineage Europe 3 (Germany, 2011) in *Culex pipiens* biotype *molestus* (experiment #6); Table S4: Viral loads in the mosquito bodies, legs and wings and saliva, and the corresponding amount of viral RNA in the mosquito excreta collected from the 1st, 5th or 7th day post infection (dpi) with WNV lineage 2 (Germany, 2018) in *Culex pipiens* biotype *molestus* (experiment #7); Table S5: Dependence of the amount of viral RNA in the excreta on the mosquito infection status in *Cx. pipiens* biotype *molestus*; Table S6: Correlation between the amount of viral RNA in the excreta shed from the 1st, 5th or 7th day post infection (dpi) and the viral loads in the mosquito bodies; Table S7: Correlation between the amount of viral RNA in the excreta shed from the 1st, 5th or 7th day post infection (dpi) and the viral loads in the mosquito legs and wings.
