# Supplementary material for: Excretion Dynamics of Arboviruses in Mosquitoes and the Potential Use in Vector Competence Studies and Arbovirus Surveillance
Source: Trop Med Infect Dis. 2023 Aug 11;8(8):410. doi: 10.3390/tropicalmed8080410 (PMC10459540; doi:10.3390/tropicalmed8080410)
Supplement: Supplementary file 1 [file tropicalmed-08-00410-s001.zip › tropicalmed-2507168-supplementary.pdf]

Supplementary Materials

Excretion Dynamics of Arboviruses in Mosquitoes and the Potential Use in Vector Competence Studies and Arbovirus Surveillance

Christin Körsten [1], Ana Vasić [1,2], Amira A. AL-Hosary [1,3], Birke A. Tews [1], Cristian Răileanu [1], Cornelia Silaghi [1], Mandy Schäfer [1]

[1] Institute of Infectology, Friedrich-Loeffler-Institut, Federal Research Institute for Animal Health, Greifswald-Insel Riems, 17493 Greifswald, Germany

[2] Scientific Institute of Veterinary Medicine of Serbia, 11000 Belgrade, Serbia

[3] Department of Animal Medicine (Infectious Diseases), Faculty of Veterinary Medicine, Assiut University, Assiut 71526, Egypt

Table S1. Feeding and survival rates of *Culex pipiens* biotype *molestus* (CxM) and *Aedes vexans* (AeV) in the infection experiments.

Experiment no.	Virus	Mosquito species	Feeding rate[1] % (n/n)	Survival rate[2] % (n/n)
#1	WNV lineage 1 (Italy, 2008)	AeV	25.77 (42/163)	43.59 (17/39)[4]
#2	WNV lineage 1 (Italy, 2008)	CxM	21.62 (40/185)	14 dpi: 57.89 (22/38)<br>20 dpi: 5.26 (2/38)
#3	WNV lineage 2 (Germany, 2018)	AeV	54.55 (30/55)[3]	53.33 (8/15)
#4	TBEV Western Neudörfl	CxM	25.78 (33/128)	77.42 (24/31)
#5	USUV lineage Europe 3 (Germany, 2016)	CxM	47.86 (67/140)[3]	51.43 (18/35)
#6	USUV lineage Europe 3 (Germany, 2011)	CxM	37.63 (35/93)	48.48 (16/33)
#7	WNV lineage 2 (Germany, 2018)	CxM	23.66 (22/93)	70.00 (14/20)

[1] Proportion of engorged females among all the living female mosquitoes that were offered the infectious blood meal.

[2] Proportion of mosquitoes still alive at the 14th or 20th day after infection (dpi) among all the mosquitoes that were incubated. Mosquitoes that were accidentally killed during the incubation are not included.

[3] Not all engorged females were used for the investigation of the mosquito excreta.

[4] Not all the surviving mosquitoes were dissected and examined.

Table S2. Viral loads in the mosquito bodies, legs and wings and saliva, and the corresponding amount of viral RNA in the mosquito excreta collected from the 1st or 7th day post infection (dpi) with USUV lineage Europe 3 (Germany, 2016) in *Culex pipiens* biotype *molestus* (experiment #5).

**Mosquito no.**	**Viral load in body[1]**	**Viral load in legs/wings[1]**	**Viral load in saliva[1]**	**Detection of infectious virus in saliva**	**Infection status**	**Viral RNA in excreta[1]**	
						**From 1st dpi**	**From 7th dpi**
#1	N/A	N/A	N/A	Negative	No infection	N/A	N/A
#2	N/A	N/A	N/A	Negative	No infection	1.749	N/A
#3	N/A	N/A	N/A	Negative	No infection	2.313	1.995
#4	N/A	N/A	N/A	Negative	No infection	2.156	2.170
#5	N/A	N/A	N/A	Negative	No infection	2.057	N/A
#6	N/A	N/A	N/A	Negative	No infection	1.837	N/A
#7	6.540	N/A	N/A	Negative	Infection	4.305	4.167
#8	N/A	N/A	N/A	Negative	No infection	2.423	N/A
#9	N/A	N/A	N/A	Negative	No infection	1.841	N/A
#10	N/A	N/A	N/A	Negative	No infection	1.726	N/A
#11	N/A	N/A	N/A	Negative	No infection	2.374	1.999
#12	N/A	N/A	N/A	Negative	No infection	2.353	N/A
#13	N/A	N/A	N/A	Negative	No infection	1.640	N/A
#14	N/A	N/A	N/A	Negative	No infection	2.343	N/A
#15	N/A	N/A	N/A	Negative	No infection	2.262	N/A
#16	N/A	N/A	N/A	Negative	No infection	1.687	1.633
#17	N/A	N/A	N/A	Negative	No infection	3.114	N/A
#18	N/A	N/A	N/A	Negative	No infection	1.906	1.773
#19	N/A	N/A	N/A	Negative	No infection	2.399	N/A
#20	N/A	N/A	N/A	Negative	No infection	1.702	1.702
#21	N/A	N/A	N/A	Negative	No infection	N/A	N/A
#22	N/A	N/A	N/A	Negative	No infection	N/A	N/A
#23	N/A	N/A	N/A	Negative	No infection	1.953	N/A
#24	N/A	N/A	N/A	Negative	No infection	1.832	1.723

[1] In the equivalent to log $TCID_{50}$/ml.

Table S3. Viral loads in the mosquito bodies, legs and wings and saliva, and the corresponding amount of viral RNA in the mosquito excreta collected from the 1st, 5th or 7th day post infection (dpi) with USUV lineage Europe 3 (Germany, 2011) in *Culex pipiens* biotype *molestus* (experiment #6).

Mosquito no.	Viral load in body[1]	Viral load in legs/ wings[1]	Viral load in saliva[1]	Detection of infectious virus in saliva	Infection status	Viral RNA in excreta[1]		
						From 1st dpi	From 5th dpi	From 7th dpi
#1	7.022	2.376	N/A	Negative	Disseminated infection	5.171	5.171	5.205
#2	6.412	1.871	1.616	Positive	Potential transmission	4.212	4.294	4.400
#3	1.575	N/A	N/A	Negative	Infection	1.873	1.623	1.682
#4	1.760	N/A	N/A	Negative	Infection	1.853	1.722	1.722
#5	N/A	--[2]	--	--	No infection	1.715	1.403	1.403
#6	N/A	--	--	--	No infection	1.857	1.857	2.145
#7	6.101	--	--	--	Infection	3.498	4.041	4.292
#8	7.731	6.270	3.321	Positive	Potential transmission	4.010	4.225	4.351
#9	6.675	5.453	1.804	Negative	Potential transmission	4.613	4.746	4.741
#10	7.554	5.571	1.873	Negative	Potential transmission	3.996	4.442	4.477
#11	2.176	3.088	2.134	Negative	Potential transmission	2.185	2.064	2.231
#12	2.741	2.208	N/A	Negative	Disseminated infection	2.067	2.067	2.067
#13	6.166	2.429	2.260	Negative	Potential transmission	4.033	4.529	4.509
#14	1.693	N/A	N/A	Negative	Infection	1.653	1.653	1.653
#15	6.642	2.121	1.789	Negative	Potential transmission	4.582	4.831	4.819
#16	6.503	4.959	3.016	Positive	Potential transmission	3.946	4.515	4.530
#17	1.667	N/A	N/A	Negative	Infection	1.942	2.264	2.264
#18	1.549	3.094	1.884	Negative	Potential transmission	1.686	1.621	1.751
#19	5.862	2.910	1.467	Negative	Potential transmission	3.556	3.763	3.760

[1] In the equivalent to log $TCID_{50}$/ml.

[2] The viral load in the legs/wings and saliva was not determined in mosquitoes that died before the 14th dpi.

Table S4. Viral loads in the mosquito bodies, legs and wings and saliva, and the corresponding amount of viral RNA in the mosquito excreta collected from the 1st, 5th or 7th day post infection (dpi) with WNV lineage 2 (Germany, 2018) in *Culex pipiens* biotype *molestus* (experiment #7).

Mosquito no.	Viral load in body[1]	Viral load in legs/ wings[1]	Viral load in saliva[1]	Detection of infectious virus in saliva	Infection status	Viral RNA in excreta[1]		
						From 1st dpi	From 5th dpi	From 7th dpi
#1	7.766	6.152	5.257	Positive	Potential transmission	4.833	5.620	5.620
#2	7.444	N/A	N/A	Negative	Infection	4.479	4.653	4.938
#3	3.396	3.125	4.864	Positive	Potential transmission	2.577	2.577	2.577
#4	2.873	N/A	N/A	Negative	Infection	N/A	N/A	N/A
#5	6.675	6.172	N/A	Negative	Disseminated infection	3.885	4.119	4.243
#6	7.041	5.512	4.957	Positive	Potential transmission	5.169	5.220	5.350
#7	8.165	6.693	5.659	Positive	Potential transmission	4.512	4.791	4.821
#8	7.389	--[2]	--	--	Infection	4.182	4.449	4.443
#9	7.168	5.995	5.582	Positive	Potential transmission	4.798	4.992	4.856
#10	4.367	4.42	5.378	Negative	Potential transmission	N/A	N/A	N/A
#11	6.514	5.869	5.138	Positive	Potential transmission	4.595	4.599	4.599
#12	3.274	4.662	N/A	Negative	Disseminated infection	2.813	2.813	2.813
#13	N/A	N/A	N/A	Negative	No infection	3.718	3.718	3.718
#14	N/A	N/A	N/A	Negative	No infection	3.081	3.081	3.081
#15	N/A	N/A	N/A	Negative	No infection	N/A	N/A	N/A

[1] In the equivalent to log $TCID_{50}$/ml.

[2] The viral load in the legs/wings and saliva was not determined in mosquitoes that died before the 14th dpi.

Table S5. Dependence of the amount of viral RNA in the excreta on the mosquito infection status in *Cx. pipiens* biotype *molestus*. Mean values from the excreta collected from 1st, 5th and 7th day post infection (dpi) were used. For the contrast "infection vs. no infection", data from mosquitoes that died after the 12th dpi were included. Calculations were performed with student's t-test and the Mann–Whitney rank sum test, respectively. A statistical difference was assumed at $p \leq 0.05$ *.

Virus	Contrast	*p* value
WNV lineage 2 (Germany, 2018)	Infection vs. no infection; from 1st dpi	0.218
	Infection vs. no infection; from 5th dpi	0.218
	Infection vs. no infection; from 7th dpi	0.218
	Disseminated infection vs. no disseminated infection; from 1st dpi	0.184
	Disseminated infection vs. no disseminated infection; from 5th dpi	0.168
	Disseminated infection vs. no disseminated infection; from 7th dpi	0.186
	Potential transmission vs. no potential transmission; from 1st dpi	0.097
	Potential transmission vs. no potential transmission; from 5th dpi	0.128
	Potential transmission vs. no potential transmission; from 7th dpi	0.209
USUV lineage Europe 3 (Germany, 2011)	Infection vs. no infection; from 1st dpi	0.127
	Infection vs. no infection; from 5th dpi	0.097
	Infection vs. no infection; from 7th dpi	0.127
	Disseminated infection vs. no disseminated infection; from 1st dpi	**0.006 ***
	Disseminated infection vs. no disseminated infection; from 5th dpi	**0.034 ***

Disseminated infection vs. no disseminated infection; from 7th dpi	**0.013 ***
Potential transmission vs. no potential transmission; from 1st dpi	**0.049 ***
Potential transmission vs. no potential transmission; from 5th dpi	**0.034 ***
Potential transmission vs. no potential transmission; from 7th dpi	**0.027 ***

Table S6. Correlation between the amount of viral RNA in the excreta shed from the 1st, 5th or 7th day post infection (dpi) and the viral loads in the mosquito bodies. Calculations were performed with the Pearson product–moment calculation. Statistical significance was assumed at $p \leq 0.05$ *. A strong correlation was assumed at an r value of > 0.5 ***; a medium correlation was assumed at an r value from 0.1 – 0.5 **; and a weak correlation was assumed at an r value of < 0.1 *.

Virus	Tested time period	*p* value	*r* value
WNV lineage 2 (Germany, 2018)	From 1st day	**0.0166 ***	**0.626 *****
	From 5th day	**0.0110 ***	**0.655 *****
	From 7th day	**0.0097 ***	**0.662 *****
USUV lineage Europe 3 (Germany, 2011 and Germany, 2016)	From 1st day	**≤ 0.001 ***	**0.951 *****
	From 5th day	**≤ 0.001 ***	**0.963 *****
	From 7th day	**≤ 0.001 ***	**0.967 *****

Table S7. Correlation between the amount of viral RNA in the excreta shed from the 1st, 5th or 7th day post infection (dpi) and the viral loads in the mosquito legs and wings. Calculations were performed with the Pearson product–moment calculation. Statistical significance was assumed at $p \leq 0.05$ *. A strong correlation was assumed at an r value of > 0.5 ***; a medium correlation was assumed at an r value from 0.1 – 0.5 **; and a weak correlation was assumed at an r value of < 0.1 *.

Virus	Tested time period	*p* value	*r* value
WNV lineage 2 (Germany, 2018)	From 1st day	0.0663	0.504
	From 5th day	0.0537	0.525
	From 7th day	0.0620	0.511

USUV lineage Europe 3 (Germany, 2011 and Germany, 2016)	From 1st day	0.0822	0.521
	From 5th day	0.0685	0.542
	From 7th day	0.0592	0.558

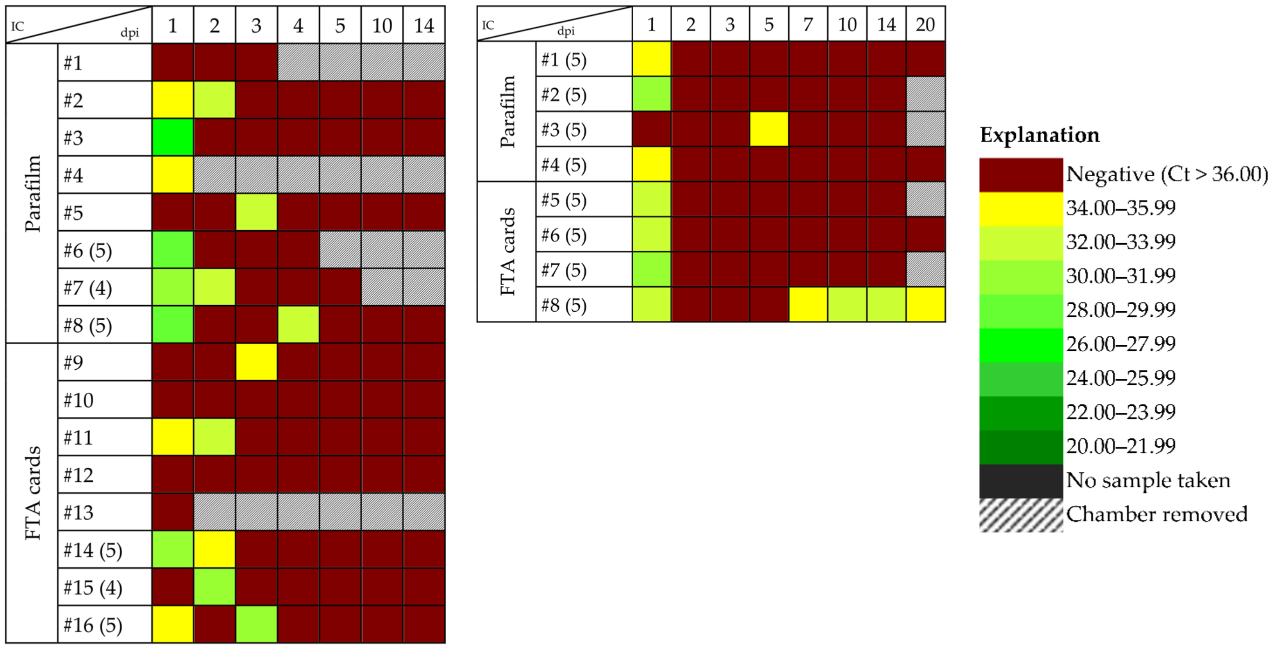

(a) (b) (c)

Figure S1. Comparison between the methods for collecting mosquito excreta. Mosquitoes from the same population were infected and excreta were collected on either Parafilm or FTA cards during the entire incubation period. Colors indicate the measured Ct values in the mosquito excreta collected from each incubation chamber (IC) during (a) experiment #1 (WNV lineage 1 in *Ae. vexans*) and (b) experiment #2 (WNV lineage 1 in *Cx. pipiens* biotype *molestus*). Corresponding Ct values are shown in (c). Numbers in brackets after the IC number indicate the total numbers of individuals in this IC if there was more than one individual.

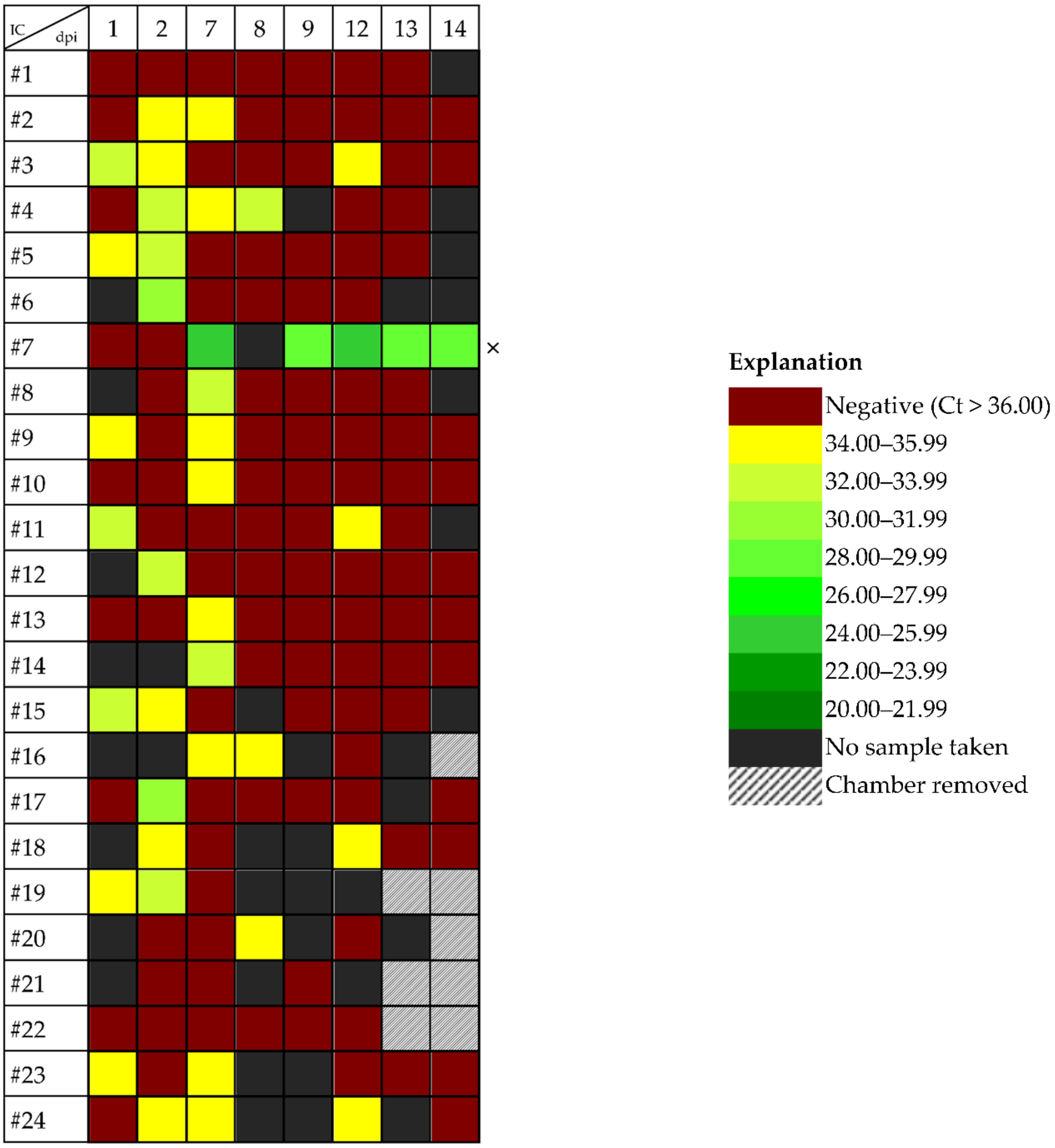

(a) (b)

Figure S2. Excretion of viral RNA after infection with USUV lineage Europe 3 (Germany, 2016) at a low titer (experiment #5; a). Only data from mosquitoes that survived the 12th day post infection (dpi) are shown. The infection status of the individual mosquitoes is indicated as infection (×), disseminated infection (××) and potential transmission (×××). (b) Colors indicate the measured Ct values in the mosquito excreta collected from each incubation chamber (IC).
